# Correlation between Pesticide Resistance and Enzyme Activity in the Diamondback Moth, *Plutella xylostella*

**DOI:** 10.1673/031.013.13501

**Published:** 2013-11-26

**Authors:** Ya-Jun Gong, Ze-Hua Wang, Bao-Cai Shi, Zong-Jiang Kang, Liang Zhu, Gui-Hua Jin, Shu-Jun Weig

**Affiliations:** Institute of Plant and Environmental Protection, Beijing Academy of Agriculture and Forestry Sciences, Beijing 100097, China

**Keywords:** acetylcholine esterase, bioassay, carboxylesterase, glutathione S-transferase

## Abstract

The diamondback moth, *Plutella xylostella* (L.) (Lepidoptera: Plutellidae), is one of the most important pests that has developed high pesticide resistance. The resistances of five Chinese populations of this moth, four resistant strains (from Beijing, Henan, Fujian, and Guangdong) and one susceptible strain, to five pesticides were determined, and the activities of carboxylesterase, glutathione S-transferase, and acetylcholine esterase were tested in all five populations. The correlations between pesticide resistance and enzyme activity were analyzed. The results showed that the resistance status to the five pesticides was different among the five populations. The resistance ratios of the Beijing and Henan populations to spinosad were 5.84 and 8.22, respectively, and those to beta-cypermethrin were 4.91 and 4.98, respectively. These ratios were higher than those for the Fujian and Guangdong populations. The Fujian population was more sensitive to abamectin and chlorpyrifos than the susceptible population (the resistance ratios were 0.14 and 0.91, respectively); in fact, the median lethal concentration for *P. xylostella* was significantly higher for chlorpyrifos than that for any of the other four pesticides. The carboxylesterase activity in *P. xylostella* showed positive correlations with the resistance to spinosad, beta-cypermethrin, chlorpyrifos, and abamectin, but no correlation was observed between the carboxylesterase activity and resistance to emamectin benzoate, between glutathione S-transferase activity and resistance to any of the five pesticides tested, or between acetylcholine esterase activity and any of the pesticides except for emamectin benzoate.

## Introduction

The diamondback moth, *Plutella xylostella* (L.) (Lepidoptera: Plutellidae) is a major pest of cruciferous vegetables worldwide. In the tropics and subtropics, where cruciferous plants are grown year-round, this pest can be present at any time (Talekar and Shelton 1993). Due to the fast population growth of *P. xylostella* with severe generational overlap, this pest can seriously damage vegetable production. Combined management costs and yield losses due to *P. xylostella* are estimated to be $4–5 billion USD annually worldwide (Furlong et al. 2013). There are two characteristics that make *P. xylostella* one of the most cosmopolitan pests: rapid development of resistance to multiple pesticides and an ability to migrate and disperse over long distances (Mackenzie 1958; Chu 1986; Talekar and Shelton 1993; Tabashnik 1994; Chapman et al. 2002; Chapman et al. 2003). Among various control methods, application of synthetic insecticides is overwhelmingly the most common control strategy (Grzywacz et al. 2010). To control *P. xylostella*, farmers often increase the pesticide concentrations, increase the frequency of application, and mix various pesticides together. Unfortunately, these activities also support the development of serious pesticide resistance in these moths. To date, populations of *P. xylostella* have generated resistance to every pesticide used extensively against them, including organophosphates, carbamate, pyrethroid, antibiotics, and biological agents (Bt) (Talekar and Shelton 1993; Tabashnik 1994). To complicate matters, the migration of *P. xylostella* leads to unstable population structures and difficulty in monitoring the status of pesticide resistance (Caprio and Tabashnik 1992; Coulson et al. 2002; Kim et al. 2003; Wang et al. 2005; Endersby et al. 2006; Li et al. 2006; Saw et al. 2006).

The resistance of *P. xylostella* to insecticides is primarily a function of increased activity of metabolic enzymes (Hung and Sun 1989), decreased cuticular penetration (Noppun et al. 1989), insensitivity of the target site (Konno and Shishido 1994), decreased nerve sensitivity, and knockdown resistance (Hama et al. 1987; Kwon et al. 2004).

Metabolic capacity is strongly related to the activities of the enzymes carboxylesterase (CarE), glutathione S-transferase (GST) (Hung and Sun 1989; Kao and Sun 1991), and cytochrome P450 monooxygenases (MFO) (Li et al. 2007; Eziah et al. 2009). Insensitivity of the target site to organophosphates and pyrethroids is usually related to the activity of acetylcholine esterase (AChE) (Baek et al. 2005; Lee et al. 2007). The correlations between pesticide resistance and enzyme activity have been widely studied. Esterases play important roles in the metabolism of organophosphates and pyrethroids (Tang and Zhou 1993). GSTs have also been reported to be associated with resistance of *P. xylostella* to organophosphates (Kao et al. 1989; Tang et al. 1992; Wu et al. 2000), abamectin, and β- cypermethrin (Pei et al. 2003).

However, Li et al (2000) demonstrated no close correlation between resistance and MFO activity (Li et al. 2000). Zhuang et al. (2001) indicated that an increase in temperature would inhibit the activities of AChE, CarE, and GST, reducing the pesticide resistance of *P. xylostella* (Zhuang et al. 2011). Consequently, variations in enzyme activities among populations of this species might result from a combination of pesticide resistance, temperature and regional differences, and other factors.

In this study, the resistance status of five different *P. xylostella* populations to five different pesticides was tested and the activities of three enzymes in each population were assayed, aiming to find correlations between pesticide resistance and the enzyme activities and the impact of regional factors on enzyme activity.

## Materials and Methods

### Insects

Insects Because *P. xylostella* has proven to be a migratory pest, populations in northern China, where it becomes too cold for the moths to remain outside throughout winter, are likely to be composed of several different migratory populations from the southern regions (Li et al. 2006). The populations used in our study were therefore purified by multi-generational rearing in the laboratory over many years. The Fujian and Guangdong populations were taken from the Institute of Vegetables and Flowers at the Chinese Academy of Agricultural Sciences. The Guangdong population was collected from Guangzhou of Guangdong province and reared continuously for four years. The Fujian population was collected from Fuzhou of Fujian province and reared continuously for eight years. The Beijing population was collected from Mentougou and reared in our lab for four years. The Henan population was collected from Jiyuan of Henan province and reared continuously for four years. The pesticide-susceptible population was collected from Shenzhen of Guangdong province and reared in the laboratory for 20 years without any exposure to pesticides. All populations were maintained under conditions of 25° C with 75% RH and a 16:8 L:D photoperiod. Second instar *P. xylostella* larvae were selected at random for biological and enzyme activity assays.

### Pesticides

The pesticides used in this study were the following: chlorpyrifos (organophosphorus insecticide), 95% active compound (Chengdu Kelilong Biochemical, www.cdklls.cn.zhongsou.net); betacypermethrin (pyrethroid insecticide), 95% active compound (Chengdu Kelilong Biochemical); abamectin (naturalyte insecticide), 94% active compound; emamectin benzoate (naturalyte insecticide), 74.6% active compound (Jiamusi Xingyu Biotechnology Development, www.xybiochem.com); and spinosad (naturalyte insecticide), 48% suspension (Dow AgroSciences, www.dowagro.com).

### Bioassays

All pesticides were tested using the leaf-dip bioassay method. The active compounds were first dissolved in acetone to a certain technical concentration, then diluted serially to produce seven pesticide solutions of concentrations at a geometric or equal difference ratio (containing 0.1% Triton X-100). Cabbage leaves were immersed into the pesticide solutions for 10 sec and then removed to dry naturally indoors. The prepared leaves were put into Petri dishes (10 cm in diameter) with the adaxial leaf surface up. A calligraphy brush was used to move the 2^nd^ instar larvae onto the pesticidetreated cabbage leaves, 20 larvae per dish. Each of these experiments were repeated four times using larvae on solvent-treated leaves as controls. The Petri dishes were covered by Parafilm® “M” Laboratory Film (Bemis Company, www.parafilm.com) with 15–20 pinpricked holes and then placed in an incubator at 25 ± 1° C with 75% RH and a 16:8 L:D photoperiod. After 24 hr, the deaths of the tested individuals were determined on the basis of color and posture changes by touching the larvae with calligraphy brush tips. The non-moving larvae were considered to be dead.

### Enzyme activity assay

During the process of enzyme preparation, 100 of the 2^nd^ instar larvae were randomly picked from each population. Each larva was put into a 1.5 mL tube with 200 µL of 0.04 mol precooled phosphate buffer (pH = 7.0). Under ice bath conditions, the homogenate was obtained and then centrifuged at 3500 rpm at 4° C using a 5417R centrifuge (Eppendorf, www.eppendorf.com). The supernatant was taken into a new tube for the enzyme activity test.

The CarE activity test was conducted using the method of van Asperen (1962) and Zhu and Gao (1999). First, 135 µL of 0.3 mmol α- naphthyl acetate and 15 µL of enzyme preparation were added into the sample holes of a 96-well microplate and then incubated at 37° C for 30 min. Fifty µL of DBLS (prepared with 1% fast blue B salt solution and 5% sodium dodecyl sulfate at a ratio of 2:5) were used to terminate the reaction in each well. The mixture was kept at room temperature for 15 min. The production of α-naphthol as a final product was determined at 600 nm using a SpectraMax Plus Microplate Reader (Molecular Devices, www.moleculardevices.com).

The GST activity test was conducted using a GST detection kit (Nanjing Jiancheng Bioengineering Institute, www.njjcbio.com/). One unit of activity was defined as a decrease in glutathione concentration by 1 µmol in 1 mg of tissue protein for 1 min at 37° C when the effects of non-enzymatic reactions are eliminated.

The AChE activity was determined according to the instructions with an AChE detection kit (Nanjing Jiancheng Bioengineering Institute). Acetylcholine is hydrolyzed by AChe producing acetic acid and thiocholine. Thiocholine reacts with the Ellman reagent 5,5-dithiobis-2- nitrobenzoic acid to produce the anion of 5- thio-2-nitrobenzoic acid. The increase in the latter's spectrophotometric absorbance indicates enzyme activity. One unit of AChE catalytic activity was defined as the amount of enzyme that caused the decomposition of 1 µmol of acetylcholine per 6 minutes at 37° C in 1 mg protein of tissue homogenate.

The total protein concentrations were determined using a BCA protein assay kit (Pierce Biotechnology Company, www.piercenet.com) according to the manufacturer's instructions.

### Statistical analysis

DPS software (www.dpssoft.com) was used to obtain the regression equation of toxicity, the LC50, and the 95% confidence limit. The resistance ratios were calculated as the LC_50_ of the tested population/LC_50_ of the susceptible population. The enzyme test results fell within narrow ranges for each population. Thus, in the subsequent analysis, the mean values of the enzyme activities of all tested individuals were used for the analyses of the resistance ratio correlations.

## Results

### Resistance status of *Plutella xylostella*

Compared with susceptible population, the Fujian, Guangdong, Beijing, and Henan populations of *P. xylostella* developed varying degrees of resistance to beta-cypermethrin, spinosad, and emamectin benzoate. The resistance ratios of the Fujian, Guangdong, Beijing, and Henan populations to betacypermethrin were 2.29, 3.95, 4.91, and 4.98, respectively, and to spinosad were 1.46, 3.06, 5.84, and 8.22, respectively, but to emamectin benzoate they were 1.16, 3.7, 2.71, and 2.16, respectively (Table 1). The Beijing and Henan populations had higher resistances to betacypermethrin and spinosad than the Fujian and Guangdong populations had.

**Table 1.**
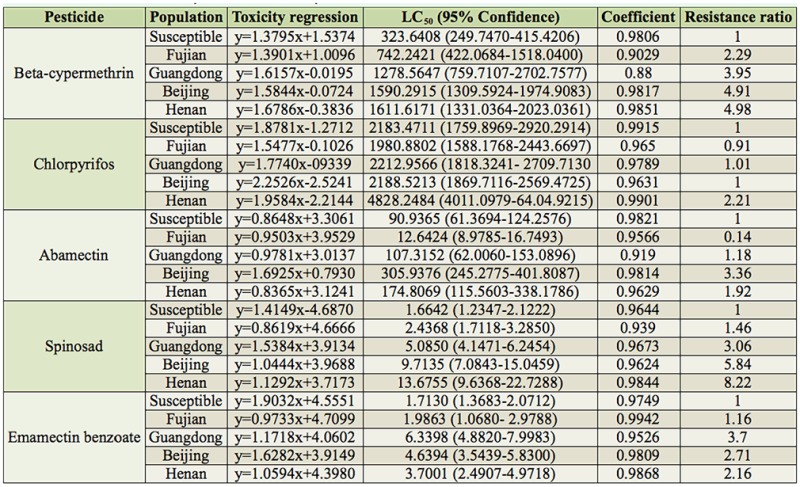
Resistance levels of *Plutella xylostella* to five pesticides.

Among the five pesticides tested, the organophosphorus chlorpyrifos had the highest LC_50_. The LC_50_ of the Henan population was up to 4828.2484 mg L_-1_, which was 2.21 times the chlorpyrifos LC_50_ of the susceptible population. The Guangdong and Beijing populations showed only small differences in resistance to chlorpyrifos compared to the susceptible population. Their LC_50_ for chlorpyrifos was 2212.9566 mg L_-1_ and 2188.5213 mg L_-1_, respectively, which was only 1.01- and 1.00-fold of the chlorpyrifos LC_50_ of the susceptible population. The Fujian population had a slightly lower LC_50_ for chlorpyrifos than the susceptible population had. Thus, these three field populations proved to be susceptible to chlorpyrifos.

Compared to the susceptible population, the Beijing population had the highest resistance to abamectin, with an LC50 of 305.9376 mg L^-1^, which is 3.36-fold that of the susceptible population, followed by the Henan and Guangdong populations with respective LC_50_ values of 174.8069 mg L^-1^ and 107.3152 mg L^-1^, which are 1.92- and 1.18-fold that of the susceptible population, respectively. The Fujian population was extremely susceptible to abamectin, and the LC_50_ was only 12.6424 mg L^-1^, 0.14-fold that of susceptible population.

The results show that *P. xylostella* developed varying sensitivity to the five pesticides, showing the greatest LC50 to chlorpyrifos followed by beta-cypermethrin, and relatively low LC50 values were observed for spinosad and emamectin benzoate. Compared to the susceptible population, the four field populations had varying resistances to betacypermethrin (the resistance ratios varied from 2.29 to 4.98), abamectin (0.14 to 3.36), spinosad (1.46 to 5.84), and emamectin benzoate (1.16 to 3.7). However, the populations showed little difference in their resistance status to chlorpyrifos.

### Enzyme activity of *Plutella xylostella*

**Figure 1.**
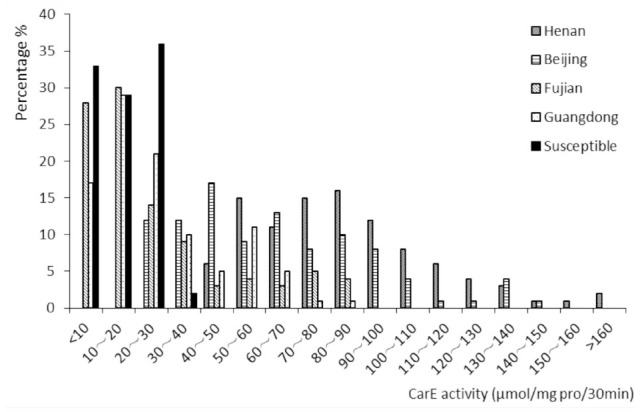
Histogram of carboxylesterase (CarE) activity offive populations of *Plutella xylostella*. High quality figures areavailable online.

Compared with the susceptible population, the Henan and Beijing populations showed significant increases in their CarE activities. Their frequencies for activity values more than 100 µmol/mg protein/30 min were 25% and 11% respectively. For activity values between 40 and 100 µmol/mg protein/30 min they were 75% and 65% respectively. And for activity values less than 40 µmol/mg protein/30 min they were 0% and 24% respectively. In the Fujian and Guangdong populations, the CarE activities were slightly higher than those of the susceptible population, and their frequencies for enzyme activity more than 100 µmol/mg protein/30 min were both 0. For activity values between 40 and 100 µmol/mg protein/30 min they were 19% and 23% respectively. And for activity values less than 40 µmol/mg protein/30 min they were 81% and 77% respectively. The enzyme activities of all individuals from the susceptible population were less than 40 µmol/mg protein/30 min (Figure 1).

**Figure 2.**
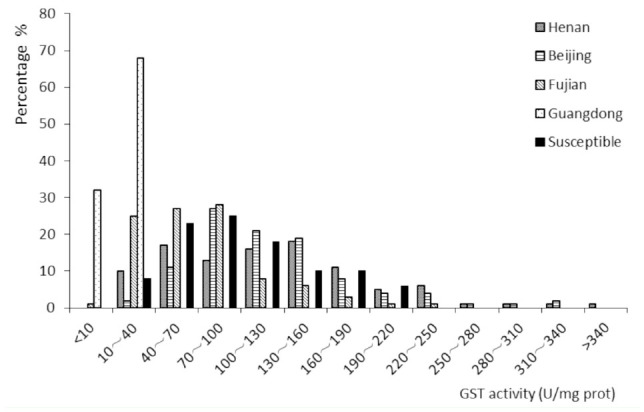
Histogram of glutathione S-transferase (GST) activity of five populations of *Plutella xylostella*. High quality figures are available online.

There were few differences in GST activity among the Henan, Beijing, and susceptible populations. The frequencies of enzymatic activities were 44%, 39%, and 26% respectively for activities greater than 130 U/mg protein; 46%, 59%, and 66% respectively for activities between 40 and 130 U/mg protein; and 10%, 2%, and 8% respectively for activities less than 40 U/mg protein. On the other hand, the Fujian population presented lower enzymatic activity when compared to the susceptible population. The activity frequencies of the Fujian population were 11%, 63%, and 26% for activity values less than 40, between 40 and 130, and more than 130 U/mg protein respectively. The Guangdong population presented remarkably lower GST activities when compared to the susceptible population, as shown clearly by the relatively large frequencies for the values of the enzymatic activity that are less than 40 U/mg protein (Figure 2).

**Figure 3.**
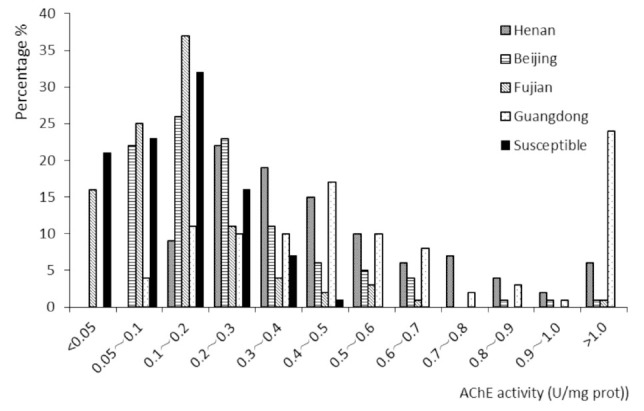
Histogram of acetylcholine esterase (AChE) activity of five populations of *Plutella xylostella*. High quality figures are available online.

Compared with the susceptible population, the Guangdong population had the most significant increase in AChE activity. The AChE activities in 48% of the population were greater than 0.5 U/mg protein, the activities in another 48% of the population were between 0.1 and 0.5 / mg protein, and the activities in the remaining 4% of the population were lower than 0.1/U mg protein. In contrast, the Henan and Beijing populations presented lower AChE activities compared to the susceptible population, as 35% of the Henan population and 12% of the Beijing population had AChE activities above 0.5 U/mg protein, 65% and 66% had activities between 0.1 and 0.5 U/mg protein respectively, and 0% and 22% respectively had activities less than 0.1 U/mg protein. The AChE activity of the Fujian population differed only slightly from that of the susceptible population (Figure 3).

**Table 2.**
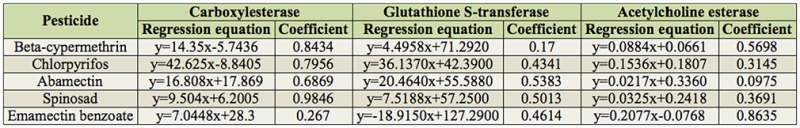
Regression analyses on the correlation of resistance ratio and enzyme activiity in *Plutella xylostela*.

### Correlation between pesticide resistance and enzyme activity

Linear regression analyses were conducted on the enzyme activities of CarE, GST, and AChE with the pesticide resistance ratios of beta-cypermethrin, chlorpyrifos, abamectin, spinosad, and emamectin benzoate. There were high positive correlations between the activity of CarE and the resistances to spinosad and beta-cypermethrin (the r-values were 0.9846 and 0.8434, respectively) (Table 2). There were correlations between the CarE activity and the resistance to chlorpyrifos and abamectin to some degree (the r-values were 0.7956 and 0.6869, respectively). There was little correlation between CarE activity and resistance to emamectin benzoate.

As shown by the distribution regions of GST activity, there was little difference among the GST activities of the Beijing, Henan, and susceptible populations, whereas for the Fujian and Guangdong populations the GST activities were markedly reduced. The linear regression analyses indicated that the GST activities had little correlation with the resistance ratios to pesticide, showing that there is no close relation between GST activity and pesticide resistance in *P. xylostella* (Table 2).

The distribution regions of AChE activity showed that the AChE activity in the Guangdong population was significantly higher than that of the susceptible population but differed only slightly between the Fujian population and the susceptible population. According to the linear regression analyses, AChE activity is related to resistance to emamectin benzoate to some degree, with a correlation coefficient of 0.8635, but AChE activity has a low correlation with other pesticide resistances (Table 2).

## Discussion

### Resistance of *Plutella xylostella* and pesticide usage in China

Pest resistance to pesticide is a hereditary characteristic that differs to some extent among individuals of the same population (Maa and Liao 2000). Because of the repeated use of pesticides, individuals with weak resistance decrease and die, while those with great resistance increase their proportion gradually. Therefore, the resistance of *P. xylostella* to different pesticides is largely related to the development history, frequency of use, and applied selection pressure for each pesticide. Since the 1980s, many regions of China have chosen organophosphorus, carbamate, and pyrethroid pesticides as the major chemicals for the prevention and treatment of *P. xylostella*, causing *P. xylostella* to generate high resistances to these three types of pesticide. The results of our study show that chlorpyrifos and beta-cypermethrin have the greatest LC50 values for *P. xylostella* among the five tested pesticides, though the highest resistance ratio was found in spinosad. All of the populations used in the study, including the susceptible population, had been selected by chlorpyrifos before collection from the field. The susceptible population's higher resistance to chlorpyrifos compared to the Fujian population might have developed before the collection of the parent generation of the susceptible population.

Abamectin, as the preferred pesticide in the 1990s for prevention and treatment of *P. xylostella*, has been widely spread and applied (Lasota and Dybas 1991) and is still used in the field in many areas (Pu et al. 2010). Among the three tested antibiotic pesticides, abamectin has been used for the longest period of time and also has the highest frequency of application. The test results show that its LC50 for the *P. xylostella* is clearly higher than those of the other antibiotic pesticides.

It should be noted that the populations used in the study were reared in the laboratory for several years, and that the resistances to some pesticides might have be lost during that time. Abamectin has been used for controlling *P. xylostella* only since the 1990s, so several populations used in this study might never have been exposed to abamectin previously. Thus, the varying pre-existing sensitivities of the populations to abamectin might explain why the resistance level of the Fujian population was lower than that of the susceptible population.

### Resistance ratio and susceptible population

A resistance ratio is a relative index related to the pesticide sensitivity of the susceptible population. In 1998, abamectin had generated 9 to 32 times greater resistance after application for two years in several areas of Yunnan Province of China (Zhang and He 1998). Feng and Chen (2001) reported that in the Guangdong areas of China, *P. xylostella* had an annually increasing resistance to abamectin, which was up to 20 fold in 1996. Although the resistance ratios in the above-mentioned regions were obviously higher than those found in our study, the LC50 values reported for the above populations were significantly lower than those determined in our study. The differences in the resistance ratios might be attributable to the different susceptible populations used for the ratio calculations in the different studies.

### Pesticide resistance and enzyme activity

Compared with the susceptible population, the four field populations showed some increased resistance to spinosad, beta-cypermethrin, chlorpyrifos, and abamectin, together with corresponding increases in CarE activity. Esterases are frequently implicated in the resistance of insects to organophosphates, carbamates, and pyrethroids (Hemingway and Ranson 2000, Srigiriraju et al. 2009). In a previous study, the resistances to organophosphate and indoxacarb pesticides were found to be positively correlated with an increase in esterase activity (Doichuanngam and Thornhill 1989; Sayyed and Wright 2006), which is congruent with our study. It has also been reported that an abamectinresistant strain of *P. xylostella* had notably enhanced MFO activity (Qian et al. 2008). In our study, an intermediate association between CarE activity and abamectin resistance was found. For spinosad, no relationship was reported between resistance and the activities of CarE and GST in *Helicoverpa armigera* (Wang et al. 2009). However, we found that the activity of CarE was highly correlated with resistance of *P. xylostella* to spinosad.

GSTs can mediate resistance to organophosphates, organochlorines, and pyrethroids in insects. In *P. xylostella*, GSTs have been reported to be involved in resistance to pesticides such as acephate, indoxacarb, and chlorfluazuron (Sonoda and Tsumuki 2005; Nehare et al. 2010; Sonoda and Igaki 2010). However, we found no obvious association between GST activity and resistance to any pesticide tested in this study. The Fujian and Guangdong populations were from the southern regions of China with warm climates where *P. xylostella* occurs year-round and the frequency of pesticide use is high. However, the pesticide resistances and enzyme activities were predominantly lower for these populations than for the Henan and Beijing populations. It is possible that due to the laboratory field populations' relatively long indoor feeding time and low selection pressures, some unstable resistance factors may have decreased and some pesticide sensitivity may have been restored. Regarding the enzyme, warmer climates of origin may have resulted in the reduction of enzyme activities as part of adaptation. In addition, other ecological and environmental differences may have contributed to the variations in enzyme activities among different populations. For example, increasing esterase activity was found to be roughly associated with decreasing latitude (Maa et al. 2004). The influence that geographic differences exert on pesticide selection pressures in *P. xylostella* requires further study.

An association was also found between the AChE activity and the resistance to emamectin benzoate. The frequency distribution of AChE activities was the same as the distribution of changes in resistance to emamectin benzoate. A previously reported genetic analysis indicated that a transient upregulation of immune and metabolic statuses in *P. xylostella* that were tolerant of emamectin benzoate could be transmitted to offspring by a maternal effect (Rahman et al. 2010). However, in another abamectinresistant population of *P. xylostella*, greater pesticide resistance did not significantly influence the AChE activity (Li et al. 2000). This indicates that different mechanisms must be involved in the *P. xylostella* resistance to abamectin and emamectin benzoate. Although AChE has been found to be responsible for resistance to prothiofos, other organophosphates and pyrethroids in this species (Yu and Nguyen 1996; Baek et al. 2005; Lee et al. 2007), there was no obvious correlation between AChE activity and resistance to betacypermethrin, chlorpyrifos, abamecti,,or spinosad in our study.

### Conclusions

Our research demonstrated that among the five pesticides tested, chlorpyrifos showed the highest LC50 for *P. xylostella*, followed by beta-cypermethrin. The Beijing and Henan *P. xylostella* populations had significantly higher resistances to most pesticides than did the Fujian and Guangdong populations. *Plutella xylostella* generally showed the highest resistance ratios to beta-cypermethrin and spinosad compared to the other tested pesticides. CarE activity was closely associated with pesticide resistance in *P. xylostella* but was not closely correlated with the GST and AChE activities. Pesticide resistance may also be caused by other mechanisms and factors, such as regional, genetic, and environmental differences among different populations.

## Abbreviations:

AChE,: acetylcholine esterase;
CarE,: carboxylesterase;
GST,: glutathione S-transferase;
MFO,: cytochrome P450 monooxygenases
